# Simulation-based training for endoscopic mitral valve repair: the impact on basic surgical skills for placement of sutures at mitral valve annulus during 2-h training workshop

**DOI:** 10.1093/icvts/ivae003

**Published:** 2024-01-13

**Authors:** Shokoufeh Checheili Sobbi, Yochun Jung, Marvin Fillet, Farhad Bakhtiary, Jos G Maessen, Peyman Sardari Nia

**Affiliations:** Department of Cardiothoracic Surgery, Heart and Vascular Centre, Maastricht University Medical Centre, Maastricht, Netherlands; Maastricht University, Maastricht, Netherlands; Department of Cardiothoracic Surgery, Heart and Vascular Centre, Maastricht University Medical Centre, Maastricht, Netherlands; Maastricht University, Maastricht, Netherlands; Maastricht University, Maastricht, Netherlands; Department of Cardiac Surgery, Universitatsklinikum Bonn, Bonn, Germany; Department of Cardiothoracic Surgery, Heart and Vascular Centre, Maastricht University Medical Centre, Maastricht, Netherlands; Maastricht University, Maastricht, Netherlands; Department of Cardiothoracic Surgery, Heart and Vascular Centre, Maastricht University Medical Centre, Maastricht, Netherlands; Maastricht University, Maastricht, Netherlands

**Keywords:** Mitral valve, Simulation-based training, Endoscopic

## Abstract

**OBJECTIVES:**

The aim of this study was to evaluate the impact of simulation-based training on surgical skills during 2-h learning labs during surgical annual meeting.

**METHODS:**

During the 36th European Association of Cardiothoracic Surgery annual meeting a learning drylab for simulation-based training for endoscopic mitral valve repair was set up. For this purpose, a validated high-fidelity endoscopic mitral valve surgery simulator and a validated suturing map were used. The training lasted 2 h. Technical pre- and post-assessment were carried out based on time and accuracy to place a suture at the posterior mitral valve annulus. The suture had to be placed within 60 s. The suture was considered anatomically correct if it entered and exited the annulus at the designated place (on the posterior annulus) and accurate if placed within the right width (8–12 mm).

**RESULTS:**

In total, 46 participants were included in this study, of whom 18 (38%) were experienced/staff surgeons, 23 (51%) fellows and 5 (11%) residents. Before the training, 48% of the participants failed to place any suture for pre-assessment. After completing the training, 100% of the participants succeeded in placing an anatomically correct suture. There was a significant improvement in the time taken [pre-assessment mean 45 (standard deviation: 25) s vs post-assessment mean 18 (standard deviation: 12) s, *P* < 0.001] and the accuracy to place a suture in the mitral valve annulus after completing the training (pre-assessment 32.6% vs post-assessment 65.2%, *P* < 0.001).

**CONCLUSIONS:**

This study shows a significant improvement in endoscopic skills for mitral valve surgery after completing a 2-h training with a high-fidelity endoscopic mitral valve surgery simulator. This suggests that simulation trainings during scientific annual meetings are effective on surgical skills.

**Figure:**
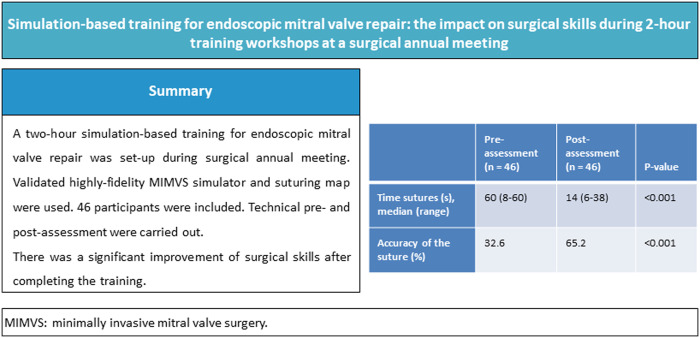

## INTRODUCTION

Scientific annual meetings of medical organizations and professional societies play an essential role in lifelong learning, exchanging insights, networking and career development [1]. European Association of Cardiothoracic Surgery (EACTS) annual meeting is the largest cardiothoracic meeting in the world [2, 3]. Exhibitions and learning labs at this meeting play an important role in keeping up to date with new innovations and developments. Participants could practice different surgical skills during hands-on workshops and learning labs. Surgical simulation training has been shown to be important in surgical education [4–7]. Therefore, the use of simulation-based training at workshops has gained interest and is rapidly increasing in the past few years [8]. However, these simulation-based workshops are associated with high costs and manpower for funders, organizers and participants. While some simulators have been investigated for their validity and the effectiveness in learning skills during dedicated multiple day courses, the effectiveness during a few hours workshop during annual meetings has not been studied before.

We have developed a high-fidelity minimally invasive mitral simulator and validated the platform before [9]. Furthermore, we have validated the educational value of our simulator in the 2 days of endoscopic port-access mitral valve repair simulation training and showed that it significantly improves endoscopic skills [4]. The aim of this study was to study the effectiveness of simulation-based training on surgical skills at short learning labs.

## METHODS

### Study design

**Figure 1:**
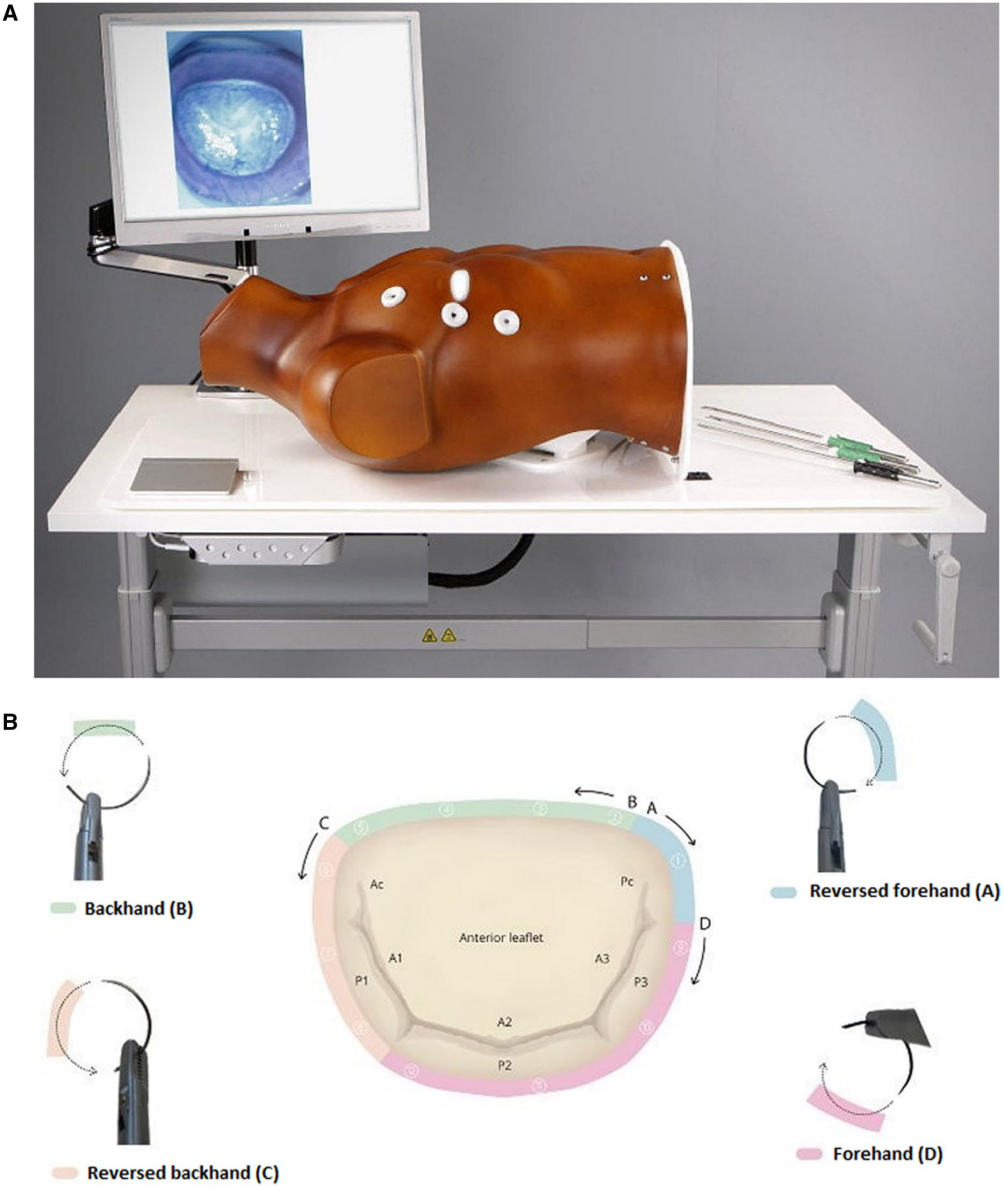
(**A**) High-fidelity minimally invasive mitral valve surgery simulator. (**B**) Suturing map for endoscopic mitral valve repair.

During 36th EACTS annual meeting, a learning lab for simulation-based hands-on training on endoscopic mitral valve repair was set up. This learning lab was sponsored by Medtronic (Medtronic Inc., Minneapolis, MN, USA). Medtronic provided the funding and space for the setup of the learning lab. A validated high-fidelity minimally invasive mitral valve surgery (MIMVS) simulator developed by Sardari Nia *et al.* [9] was used (Fig. 1 and Video 1). The MIMVS simulator consists of a central chest plate with openings to simulate utility port where endoscopic instruments can be inserted, a 3D-printed silicone valve and a camera which is focused on the silicone valve. The simulator provides metric-based feedback based on the measurement of suture width and depth. In addition, a validated suturing map for endoscopic mitral valve repair was provided to the participants for placement of the sutures with different needle angles along the mitral valve annulus [10]. Training lasted for 2 h during which expert peer formative feedback and instructions were given. A technical assessment was carried out before and after the training was completed. This was based on time taken and accuracy to place a suture at the posterior mitral valve annulus. The suture had to be placed within 60 s. The suture was considered anatomically correct if it entered and exited the annulus at the right place (on the posterior annulus) and accurate if placed within the right width (8–12 mm) measured by the high-fidelity objective metric-based feedback algorithm.

### Ethics

All participants signed an informed consent to use their data anonymously for this study.

### Statistical analysis

Data were analysed based on a normal data distribution. Continuous variables were presented as frequencies and percentages, mean with standard deviation or median and the 25th and 75th percentiles in the presence of skewness. Categorical variables were presented as frequencies and percentages. Paired *t*-tests for continuous normally distributed data, Wilcoxon signed rank tests for continuous non-parametric data, McNemar’s test for categorical data or one-way analysis of variance test for differences between the groups were used to compare the results. *P*-values <0.05 were considered statistically significant.

## RESULTS

In total, 46 participants were included in this study. The study population consisted of 18 (38%) experienced/staff cardio (thoracic) surgeons, 23 (51%) fellows and 5 (11%) residents in cardiac surgery. The results of technical skills by pre- and post-assessments are depicted in Table 1. Before the training, 24 (48%) participants failed the assessment (could not place any suture within 60 s on mitral valve annulus). After completing the training, 46 (100%) participants succeeded in placing an anatomically correct suture. There was a significant improvement in the speed to place a suture [pre-assessment mean 45 (standard deviation: 25) s vs post-assessment mean 18 (standard deviation: 12) s, *P* < 0.001] and the accuracy of sutures (pre-assessment 32.6% vs post-assessment 65.2%, *P* < 0.001) after completing the training.

**Table 1:** A comparison of technical pre- and post-assessment

	Pre-assessment (*n* = 46)	Post-assessment (*n* = 46)	*P*-value
Time sutures (s), mean (SD)	45 (25)	18 (12)	<0.001
Accuracy of the suture (%)	32.6	65.2	<0.001

SD: standard deviation.

The influence of experience could not be statistically analysed because of the small numbers of the groups.

## DISCUSSION

Large multi-day conferences often provide participants a learning experience through exhibition and workshops. Simulation-based trainings are an important part of learning labs during EACTS annual meetings. The improvement of surgical skills by simulation training has been established in various research before [4–7]. There is growing interest in participating in simulation-based workshops [11, 12]. Although most simulators have been studied during longer hands-on courses, the effect of short simulation trainings during annual meetings in cardiac surgery has not been investigated before. Earlier research shows a significant improvement in laparoscopic, robotic and suturing skills of students and residents in general surgery even after completing a simulation-based training of 30 min to 3 h [13–15]. MIMVS simulator has proven to improve endoscopic surgical skills of the participants who attend the EACTS mitral valve repair course of 2 days [4]. Our study highlights the improvement in surgical skills after completing a 2-h simulation training using a validated MIMVS simulator and a suturing map during a workshop of a duration of merely 2 h. The surgical skills of all participants with different levels of expertise were improved significantly.

The findings of this study indicate that simulation-based workshops during conferences and annual meetings could be of great value when using validated and well-studied simulators.

Our research has several limitations. First, we have no data of the retainment of surgical skills gained during a 2-h workshop compared to workshop with a longer duration. We do not know if this one-time 2-h simulation training has any impact on surgical skills during real endoscopic mitral valve repair. Ideally, the improvement of surgical skills during surgery should be analysed. Additionally, our training does not allow trainees to practice every step of endoscopic mitral valve surgery. Our 2-h training focuses solely on 1 specific surgical aspect: placing sutures on the mitral valve annulus. Future research is recommended to analyse the impact of this 2-h simulation-based training on learning a new procedure to determine how often simulation-based training is needed to achieve an improvement in surgical skills in the operating theatre.

## CONCLUSION

The surgical skills of all participants with different levels of expertise were improved significantly after 2-h simulation-based training in endoscopic mitral valve repair with a validated MIMVS simulator using a validated suturing map. The findings of this study suggest that simulation-based workshops during conferences and annual meetings could be of great value when using validated and well-studied simulators. However, future research is recommended to analyse the impact on surgical skills in the operating theatre.

## Data Availability

The data underlying this article will be shared on reasonable request to the corresponding author.

## ABBREVIATIONS

EACTS: European Association of Cardiothoracic Surgery
MIMVS: Minimally invasive mitral valve surgery
